# Development of number-space associations: SNARC effects and spatial attention in 7- to 11-year-olds

**DOI:** 10.1371/journal.pone.0212204

**Published:** 2019-03-12

**Authors:** Yun Pan, Xiaohong Han, Gaoxing Mei, Xuejun Bai, Yan Chen

**Affiliations:** 1 Key Laboratory of Basic Psychological and Cognitive Neuroscience, School of Psychology, Guizhou Normal University, Guiyang, China; 2 Department of Basic Psychology, Guizhou University of Traditional Chinese Medicine, Guiyang, China; 3 Academy of Psychology and Behavior, Tianjin Normal University, Tianjin, China; Jiangsu Normal University, CHINA

## Abstract

The spatial numerical association of response codes effect, referred to as the SNARC effect, reveals that small numbers elicit faster left than right responses, and conversely, large numbers elicit faster right responses. Here, we explored the development of this number-space association by assessing how 7-, 9-, 11-year-olds, and adults differed in spatial orienting of attention on Posner’ paradigm. Compared with the previous research, we examined how the cues would affect the level and strength of the SNARC effect in children under the different attentional conditions. Subjects made parity decisions for endogenous attention (Experiment 1) and exogenous attention (Experiment 2). The results showed that adults displayed the SNARC effect in both experiments, relatively speaking, 7- to 11-year-old Chinese children’s ability of spatial numerical association progressed gradually. With endogenous attention, the SNARC effect appeared in all age groups except for 7-year-olds for invalid cues. Compared with the endogenous attention condition, the SNARC effect was more significantly affected by cues in the exogenous attention condition. This result might be owing to the fact that the SNARC effect was not demonstrated in 7-year-olds with either valid or invalid cues. Our results suggest that the differences in the spatial orienting of attention are based on the cognitive load associated with processing number information and that this process can be affected by cues. Further, there may be cross-cultural influences on the SNARC effect, as early family training may explain the results seen in this sample of Chinese 7-year-olds. Thus, reaction times decreased with increasing age in the parity judgment task, and reaction times for valid cues were faster than for invalid cues regardless of the age group in both experiments. The SNARC effect was only present for 7-year-olds for valid cues, for endogenous attention.

## Introduction

### The SNARC effect

A complex and sophisticated understanding of numbers and their manipulation is necessary in modern society. To foster this cognitive ability and develop mathematical ability, it is critical to understand how basic number skills (e.g., number-space association) develop in children[1,2]. The SNARC (spatial-numerical association of response codes) effect refers to the phenomenon where the participant’s responses to smaller-magnitude digits (e.g., “1” or “2”) are faster for the left response than the right response, and to larger-magnitude digits (e.g.,“8” or “9”) are faster for the right response than the left response in parity judgment tasks[3–4]. The effect suggested that the psychological representation of digits is coded spatially, and such spatial cognition is automatic[3,5].

Dehaene et al.[3]suggested that parity judgment task performance was associated with the mental number line (MNL), or a left-to-right orientation of increasing numerical values based on a left-to-right reading habit. This provides further evidence for a unique connection between space and number[6]. Previous studies have also shown that reaction times become faster when a mental number-location is congruent with a response location[7]. The MNL, as a spatial explanation of the SNARC effect, originates from semantic memory of number magnitude, which depends on culturally specific reading direction[3,8–12]. Further, the SNARC effect has been observed in a wide variety of experimental settings with different numerical materials, such as single digits, two-digit numbers, negative numbers, number words, numbers in different languages, dot patterns, counting fingers, and auditory or tactile magnitude information[13–17]. The breadth of these experimental results indicated that numbers have a close relationship with SNARC effect.

### Developmental perspective

Differing SNARC effects have been found in different cultures. For example, right-to-left readers (e.g., Arabic) have a reversed SNARC effect[3]. Given these cultural and educational differences, a developmental study of the SNARC was triggered. Developmental research has demonstrated that 4-year-olds can already recognize left and right in spatial tasks[18]. Berch and colleagues[13] studied the SNARC effect in young children, and found that they showed the SNARC effect in Grade 3 (c. 9.2 years old). Further evidence suggested that one-third of children in Grade 2 also showed the SNARC effect in a Swiss sample[19], meaning that the SNARC effect is gradually formed and is even stabilized before Grade 3. Moreover, an important effect of age on the SNARC effect was found in a meta-analysis that indicated that the SNARC effect was found to increase with age from childhood to old age[20]. Van Galen and Reitsma[21] assessed the SNARC effect in 7- to 9-year-olds and adults in the Netherlands via two different tasks, namely a magnitude judgment task(in which number magnitude is essential) and a gray-box detection task(in which number magnitude is irrelevant because participants were asked to respond to the box on the right or the left of the number that may turn gray). Their results showed that the SNARC effect existed in all age groups for the magnitude judgment task, but it failed to appear before grade 3 for the gray-box detection task. These results indicate that whether numerical magnitude information is handled explicitly or not is linked to the SNARC effect. When such information was not explicitly processed, such as in the gray-box detection task[21] and the parity-judgment task[13,19], the SNARC effect did not appear until age 8 to 9. However, when numerical magnitude was explicitly processed, the SNARC effect appeared at the age of 7[21]. Imbo and Gevers[22] studied the SNARC effect in 9- and 11-year-olds, and reported that verbal encoding significantly promoted the SNARC effect in both age groups.

In terms of cultural differences, previous research suggested that Chinese children develop the ability to process digits earlier than children in other countries [17]. It has been noted that Chinese children’s ability to identify numbers is trained in early family education or at the preschool stage[23]. Chinese children in grade one (average age = 7) exhibit a significant SNARC effect [24].

### Spatial attention

Spatial attention is an important component of cognition that has a close relationship with numbers. Indeed, numerical processing widely influences spatial processing; the spatial coding of visual stimuli is fast and automatic[25–28]. For example, Fischer, Castel, Dodd, and Pratt[29] presented a number (1, 2, 8, or 9), which was not task-related, to the participants. Then, a meaningless box was presented in the left or right visual field. The participants were required to respond by pressing a button when the box appeared. When small numbers (1, 2) appeared at the fixation, participants responded more quickly to the left visual field. In contrast, when large numbers (8, 9) appeared at the fixation, responses were faster to the right visual field. The results showed that small numbers can automatically direct attention toward the left visual field, and large numbers can activate attention toward the right side of space. Casarotti[5] adopted the experimental paradigm of Fischer[29] in a temporal order judgment task that assessed whether digital processing can automatically alter the distribution of spatial attention, and they found the same results as Fischer[29].

Although Fischer[29] and Stoianov[27] identified common attentional mechanisms with respect to spatial and numeric processing in typically-developing subjects, they failed to explain these mechanisms by dividing the attentional orients component (e.g., endogenous and exogenous components). Exogenous attention refers to effects driven by the essential low-level prominence of sensory inputs[30–32]. Low-level physical properties (e.g., stimulus intensity, color, or size) may trigger an involuntary, stimulus-driven, bottom-up attentional process. In contrast, endogenous attention refers to a voluntary top-down process, initiated by internal statuses and conscious expectations for a specific object or location[33–35]. These processing mechanisms are important to the selected behavior and goals of the organism in the current environment. There exist differences between the two kinds of attention. Jonides[36] suggested that exogenous orienting was less affected by cognitive load than endogenous orienting. In other words, people were able to ignore endogenous attentional shifts but were unable to ignore exogenous attention. Exogenous attention had greater SNARC effects than endogenous attention, and expectancies about cue validity and predictive value affected endogenous orienting more obviously than exogenous orienting[37–38]. In addition, Hein, Rolke, and Ulrich[39] showed that endogenous and exogenous attention produced different effects on reaction times and accuracy, with voluntary oriented attention enhancing temporal-order more than automatically oriented attention. These results emphasize that both kinds of attentional orienting operate at different processing levels within the visual system[40–42]. Further, Berger, Henik, and Rafal and Doallo et al.[43–44] found that endogenous and exogenous attention differ significantly in the time course of their development.

Using the neuro-cognitive model of attention, it was found that endogenous attention control be exerted by interactions of the dorsal regions, and exogenous reorienting of the attentional focus was mediated by more ventral regions in the right hemisphere[45–47]. In general, endogenous and exogenous attention are two independent attention systems, with different behavioral effects, and partially distinct neural substrates[40]. While number-induced attentional biases have been found by several other labs for explained it further[48]. The attentional biases could trigger different neural activity, which is evidenced by fMRI[49] and by ERP[48,50–51]. Moreover, spatial- and numerical associations -driven attentional biases have recently been generalized to mental arithmetic[52] and documented in children (e.g., van Galen and Reitsma), adults and synesthetes[21, 53–54]. These studies indicate that the spatial orienting of attention has a close relationship with cognitive processing of numbers.

### Cue-target paradigm

When referring to cue types for endogenous and exogenous attention, there can be valid trials in which the cue reliably indicates the target location, neutral trials in which neither target nor distractor location is signaled, and invalid trials in which the cue indicates a different location than the target. The comparison among these trials, allows us to differentiate attentional-orienting benefits (i.e., performance on valid-cue trials minus neutral trials) from costs (i.e., performance on invalid-cue trials minus neutral trials). Typically, valid trials produce faster and/or more accurate responses (benefits) than neutral trials; while for invalid trials, responses are slower and/or less precise (costs). The difference between costs and benefits represents the so-called attentional cuing effect[34–35].

As mentioned above, previous studies have examined the developmental characteristics of children’s number-space associations, but have not consider visual orienting of attention[55–57]. Since endogenous and exogenous attention are two independent systems for the spatial orienting of attention [40–42], how they affect children’s number-space processing remains unclear. To investigate the impact of endogenous and exogenous attention on the processing of digits, Posner’s cueing paradigm[34–35] and the standard parity judgment[3] were used in the current experiments. To examine the SNARC effect at different stages of development, we used eight Arabic numbers (1 to 9, excluding 5). Based on our previous series of experiments[58], showing that valid trials produced faster responses than invalid trials, 75% valid trials and 25% invalid trials were used to examine the attentional cuing effect.

Previous studies have shown that numerical processing in academic contexts is often influenced by attentional factors. For example, Rousselle found that in the process of solving digital problems, children who had mathematical learning difficulties often took less attentional resources than typically developing children[59]. McLean and Hitch indicated that a positive correlation existed between children's difficulties with learning mathematics and lack of attention control[60]. These studies highlight the important relationship between spatial-numerical association of response codes and attention. Indeed, although many studies have considered how the SNARC effect applies to children, few studies have examined the influence of spatial orienting of attention on numerical processing, or how endogenous and exogenous attentional cues affect the responses of children.

Thus, the purpose of the current study was to examine age group differences in the role of endogenous attention (Experiment 1) and exogenous attention (Experiment 2) in the SNARC effect using the Posner paradigm[34–35].

In Experiment 1 we put forward two hypotheses: (a) in endogenous valid cueing, each age group would have a significant SNARC effect; (b) in endogenous invalid cueing, 9-11-year-old and adults would have a significant SNARC effect which would not be found in the 7-year-olds. In Experiment 2, we hypothesize each age group would have a significant SNARC effect in exogenous valid cueing; and we examine how the SNARC effect was present in exogenous invalid cueing for each group, whether the youngest group have would demonstrate the SNARC effect.

## Experiment 1

In Experiment 1, we examined if the SNARC effect would be affected by varying the proportion of validly cued trials in an endogenous spatial orienting task, using the Posner’s paradigm and the standard parity judgment task[3, 34–35].

### Method

#### Participants

The participants were 119 healthy right-handed individuals who fell within four age groups: 7-year-olds, 9-year-olds, 11-year-olds, and adults. Their handedness was assessed by asking them which hand was usually used to hold chopsticks for eating and a pen for writing. Children were recruited from several primary schools in China. The study was approved by the Ethics Committee of the Department of Psychology at Guizhou Normal University, and guardians provided written informed consent. The study required only basic keyboard operation skills. Researchers stopped the experiment as soon as the child did not wish to continue. Adult participants were college students who were recruited from two universities. All participants had normal or corrected-to-normal vision. Table 1 shows the demographics of the participants.

**Table 1:** Basic information about participants of each age-group.

Group	Number of participants			Age
	Male	Female	Total	Mean age (years)
7-year-old	13	14	27	7.45±0.34
9-year-old	15	12	27	8.86±0.44
11-year-old	16	18	34	11.20±0.67
Adults	16	15	31	20.80±1.30

#### Apparatus and stimuli

The procedure was implemented in E-prime software and administered using a Lenovo Laptop with a 14-inch color monitor (1024 × 768 pixels). The stimuli consisted of eight white Arabic numbers from 1 to 9, excluding 5. The digital diameter of number was approximately 5 mm, when the distance between participants and computer was approximately 47 cm, the visual angle was approximately 0.6 degree. The outer diameter of the target number was 6 mm, with the visual angle of approximately 0.7 degree when the distance between participants and computer was approximately 47 cm. The circles appearing on both sides of the screen have an inner diameter of 12 mm (1.3 degrees), and an outer diameter of 15 mm (1.6 degrees). The target appeared in the middle of the screen as white digits on a black background. The participant was seated comfortably in front of the computer screen and instructed to gaze at the center of the screen. Participants made responses by pressing the “F” and “J” keys (with the left and right hand, respectively) of a standard computer keyboard.

### Procedure

**Fig 1:**
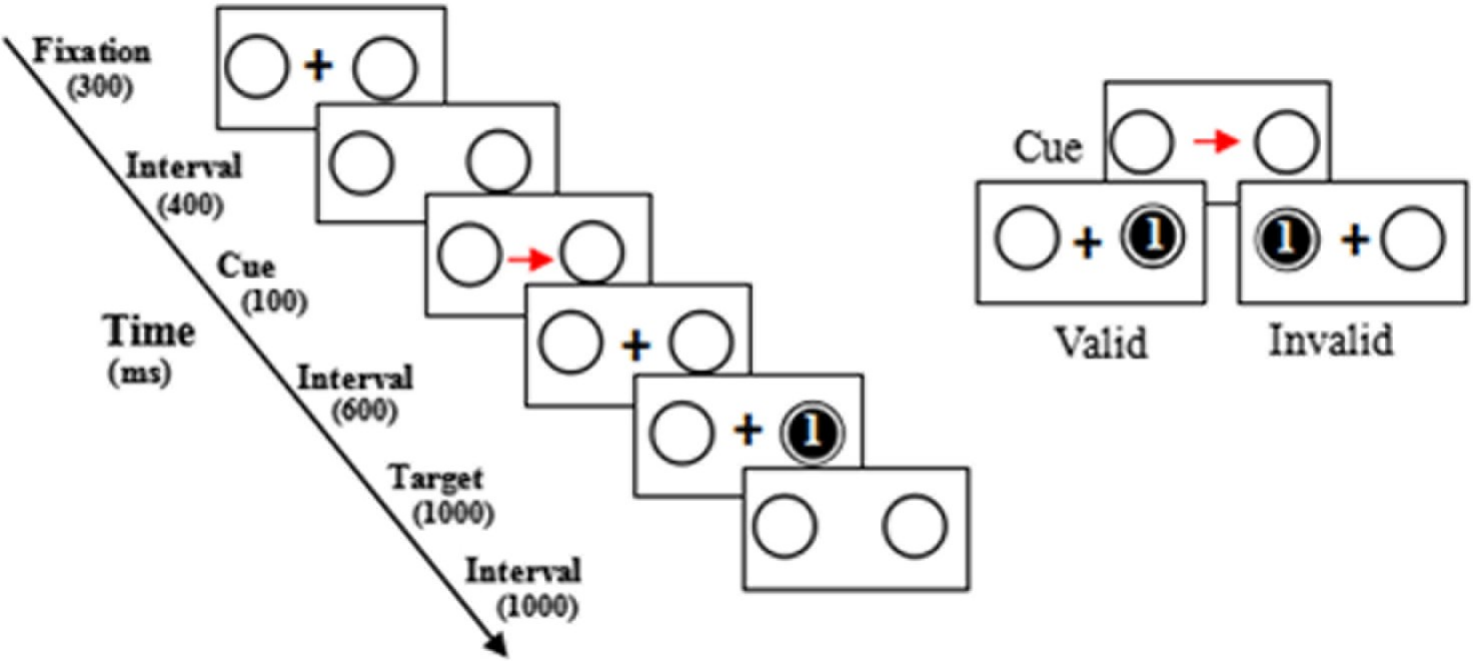
Procedure of endogenous spatial cuing task of Experiment 1.

In each trial (see Fig 1.), a fixation (“+”) was first displayed in the center of the screen for 300 ms. After a blank screen for 400 ms, a directional arrow cue was presented for 100 ms. After another blank screen of 600 ms duration, a target (i.e., a digit randomly selected from the eight digits) was displayed on the left or right side of the central fixation until the participant pressed the response key or 1000 ms had lapsed. On 75% of trials, the direction of the cue arrow was congruent with the location (left or right) of the target (valid-cue trials); for the remaining 25% trials, it was incongruent (invalid-cue trials). After another 1000 ms interval, the next trial started. Each digital stimulus was presented 24 times, 12 times on both the left and right sides. There were 18 times on the consistent side of the cue (valid), and 6 times on the side that did not coincide with the cue (invalid). There were a total of 192 trials. After 64 trials were completed, the subjects were offered a rest time. The experiment consisted of two blocks, in one block, participants pressed the left shift key for odd numbers and pressed the right shift key for even numbers, and the response keys were reversed for the other block in order to counterbalanced the two blocks. Each block began with 20 practice trials. Only when the participant obtained performance of at least 90% correct would the block-proper start.

#### Design

The experiment was composed of four independent variables: cue (two levels: valid and invalid), response hand (two levels: left or right), target magnitude (four levels: 1&2, 3&4, 6&7, 8&9), and age group (four levels: 7-year-olds, 9-year-olds, 11-year-olds, adults). To ensure all participants understood the concept of parity, especially the younger children, we used the colloquial terms dānshù (odd; literally “single number”) and shuāngshù (even; literally “double number”).

#### Statistical analyses

To examine the SNARC effect, we used two common methods: (a) an analysis of variance (ANOVA) approach[3] and (b) *t****-***tests of regression coefficients of hand differences in RTs against number magnitude[7]. A repeated-measures ANOVA was performed with the within-subjects factors of cue, response hand, and magnitude, and age group as a between-subjects factor. The results are presented in Tables 2 and 3, and based on the results we could decide the next step to analyze the SNARC effect. Next, we investigated the SNARC effect (an interaction between magnitude and hand indicated a SNARC effect in RTs) for each age group and the different cue condition. The results are shown in Table 4. Finally, we used regression analyses to further explore the strength of the SNARC effect. We subtracted the RTs of the right-hand responses from the left-hand responses for each participant, then we regressed those differences against the magnitude of the numbers to get a negative regression-coefficient which indicated the strength of the SNARC effect. The results suggested that, when the magnitude of the numbers increased from 1 to 4 and from 6 to 9, the differences in RTs (e.g., the right-hand responses minus the left-hand responses) changed from positive (e.g., faster for the left hand) to negative (e.g., faster for the right hand;) [7,14]. We then used one-sample *t*-tests to assess the significance of the mean regression coefficients (mean unstandardized *B*) of a given age group against zero. A significant *t*-value suggests a significant SNARC effect. Results are shown in Table 5.

**Table 2:** Each age-group’s mean reaction time and standard deviation (*ms*).

Age-group	The cue	Number 1&2		Number 3&4		Number 6&7		Number 8&9	
		Left hand	Right hand	Left hand	Right hand	Left hand	Right hand	Left hand	Right hand
7-year-old	Valid	739(71)	770(91)	779(88)	769(87)	778(83)	757(88)	798(69)	717(66)
	Invalid	757(85)	760(121)	777(77)	785(82)	748(142)	794(116)	803(98)	790(95)
9-year-old	Valid	679(66)	705(69)	741(78)	707(62)	737(50)	727(65)	768(81)	708(63)
	Invalid	707(74)	726(69)	760(84)	716(72)	743(78)	763(73)	798(66)	727(86)
11-year-old	Valid	638(73)	660(69)	695(62)	655(55)	684(64)	682(63)	717(55)	642(67)
	Invalid	643(71)	696(70)	701(75)	672(67)	692(73)	694(67)	734(71)	679(69)
Adults	Valid	553(69)	575(67)	598(63)	577(77)	579(68)	582(64)	637(65)	566(61)
	Invalid	565(66)	600(69)	628(75)	592(61)	585(61)	610(68)	664(69)	599(73)

**Table 3:** Results of ANOVA analyses.

Effect	*df*	*F*	*p*	*η*^*2*^_*p*_
Cue	1	34.67	0.001	0.232
Age-group	3	59.99	0.001	0.610
Cue * Age-group	3	0.45	0.716	0.012
Hand	1	17.87	0.001	0.134
Hand * Age-group	3	0.95	0.419	0.024
Magnitude	3	41.10	0.001	0.263
Magnitude * Age-group	9	2.31	0.016	0.057
Cue * Hand	1	10.09	0.002	0.081
Cue * Hand * Age-group	3	2.67	0.051	0.065
Cue * Magnitude	3	2.64	0.049	0.022
Cue * Magnitude * Age-group	9	0.53	0.856	0.014
Hand * Magnitude	3	59.35	0.001	0.340
Hand * Magnitude * Age-group	9	0.93	0.499	0.024
Cue * Hand * Magnitude	3	2.63	0.050	0.022
Cue * Hand * Magnitude * Age-group	9	1.99	0.039	0.049

A significant interaction between magnitude and age group was found, *F*(9, 345) = 2.31, *p* < .05, η^2^_p_ = 0.57, and reaction times decreased with age, as demonstrated by the difference between 7-year-olds and 11-year-olds of 93 ms. The interaction between cue and hand was significant, *F*(3, 345) = 10.09, *p* < .05, η^2^_p_ = .081, and post-hoc comparisons indicated that RTs for the right hand were shorter than those for left hand for both valid and invalid cues. The interaction between cue and magnitude was significant, *F*(3, 345) = 2.64, *p* < .05, η^2^_p_ = .022, and post-hoc comparisons indicated that RTs for valid cues were shorter than those for invalid cues for every magnitude condition. A significant interaction between magnitude and hand, *F*(3, 345) = 59.35, *p* < .001, η^2^_p_ = .340, was found, and subsequent post-hoc comparisons indicated that RTs for numbers 8 & 9 were shorter than those for 1 & 2. The interaction among cue, hand, magnitude, and age group was also significant, *F*(9, 345) = 1.99, *p* < .05, η^2^_p_ = .049.

**Table 4:** Results of ANOVA for each age-group in different cues.

Age-group	Interaction		*df*	*F*	*P*	*η2*_*p*_
7-year-old	Magnitude * Hand	Valid cue	3	8.52	0.001	0.270
		Invalid cue	3	1.32	0.277	0.072
9-year-old	Magnitude * Hand	Valid cue	3	10.84	0.001	0.294
		Invalid cue	3	7.77	0.001	0.230
11-year-old	Magnitude * Hand	Valid cue	3	22.74	0.001	0.408
		Invalid cue	3	12.14	0.001	0.269
Adults	Magnitude * Hand	Valid cue	3	21.20	0.001	0.414
		Invalid cue	3	14.40	0.001	0.324

**Table 5:** Results of one-sample t test of slope for each age-group in difference cue.

Age-group	Cue	*M*	*SD*	*t*
7-year-old	Valid	-30.97	29.13	-5.21**
	Invalid	-13.00	47.40	-1.34
9-year-old	Valid	-23.55	29.14	-4.20**
	Invalid	-20.69	33.17	-3.24*
11-year-old	Valid	-23.37	22.83	-5.79**
	Invalid	-28.23	28.98	-5.51**
Adult	Valid	-25.65	17.87	-7.99**
	Invalid	-24.08	31.05	-4.32**

Note.

* *p*<0.05

** *p*<0.01

### Results

The grand mean error rate for all participants was 8.2%, with an average error rate of 12.5% for 7-year-olds, 9.4% for 9-year-olds, 7.3% for 11-year-olds, and 3.6% for adults. Trials with incorrect responses to judgment were excluded from the analysis. Each age-group’s mean reaction times *(RTs*) and standard deviations (*SD*) are shown in Table 2.

To investigate the SNARC effect, and to further explain the four-fold interaction, a 4 (numbers) × 2 (hands) ANOVA was conducted for each age group and cue condition. An interaction between magnitude and hand indicated a SNARC effect in RTs. The results are shown in Table 4.

**Fig 2:**
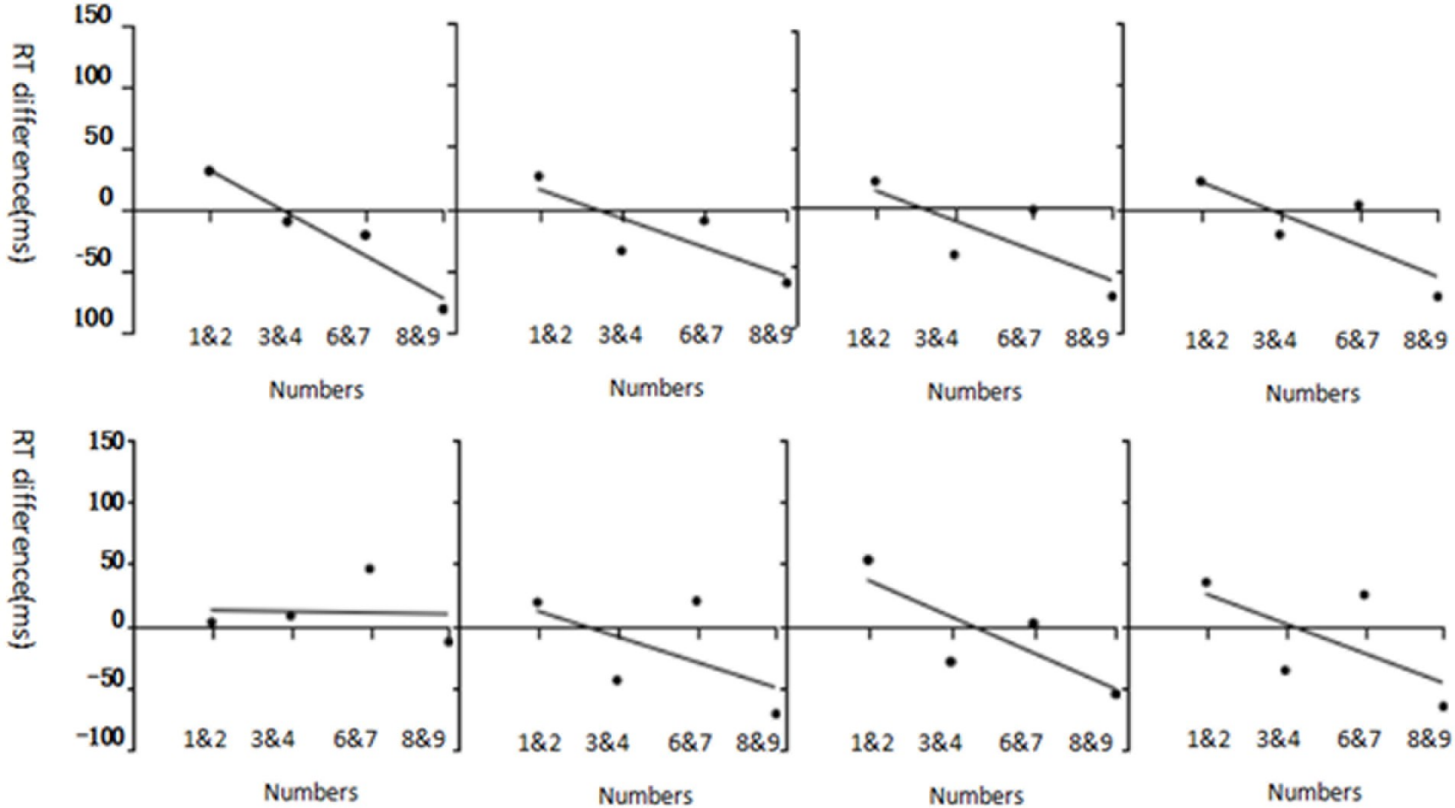
Each age group’s SNARC effect for valid cues (upper row) and for invalid cues (lower row). Panels from left to right show 7-, 9-, 11-year-olds, and adults in each row.

We then used one-sample *t*-tests to assess the significance of the mean regression coefficients (mean unstandardized *B*) of a given age group against zero to determine the strength of the cue affect and the SNARC effect. A significant *t*-value suggests a significant SNARC effect. Results are shown in Table 5. As shown in Fig 2, all age-groups showed a significant SNARC effect, except for 7-year-olds in the invalid-cue condition (*p >* .05, *t* = -1.34).

### Discussion

The results of Experiment 1 accepted both hypotheses, as each age group displayed a significant SNARC effect in endogenous valid cueing condition, and 9-, 11-year-olds and adults had a significant SNARC effect in endogenous invalid cueing condition. In addition, the experiment revealed three useful findings. The first finding was that performance in the parity task produced a pattern of data that was similar to patterns observed in other studies of endogenous spatial-orienting tasks (see Klein for a review)[61–62]. Moreover, responses were slower for invalidly cued than for validly cued trials. Second, regardless of the method used to calculate the effect, for all age-groups, the SNARC effect was sensitive to the proportion of validly cued trials. Third, and most importantly, compared with 9-,11-year-olds, and adults, a SNARC effect was only found in validly cued conditions for 7-year-old subjects, not in invalidly cued conditions, suggesting that cueing altered younger children’s responses.

## Experiment 2

In Experiment 1, we examined whether validly cued trials in an endogenous spatial orienting task would elicit a context-specific influence on the SNARC effect. Experiment 2 followed the same logic as Experiment 1, but we adopted an exogenous spatial orienting task with a shorter interval between cue and response. And we hypothesize each age group would have a significant SNARC effect in exogenous valid(or invalid) cueing; whether the youngest group have would demonstrate the SNARC effect.

### Method

#### Participants

The participants consisted of 114 healthy right-handed students from four age-groups: 7-year-olds, 9-year-olds, 11-year-olds, and adults. The selection criteria for participants were the same as in Experiment 1 and followed the ethical approval procedures. Table 6 shows the demographics of the participants.

**Table 6:** Basic information about participants of each age-group.

Group	Number of participants			Age
	Male	Female	Total	Mean age (years)
7-year-old	14	15	29	7.53±0.33
9-year-old	13	15	28	9.24±0.37
11-year-old	13	13	26	11.25±0.69
Adults	17	14	31	20.77±1.46

#### Apparatus and stimuli

These details were as in Experiment 1.

### Procedure

**Fig 3:**
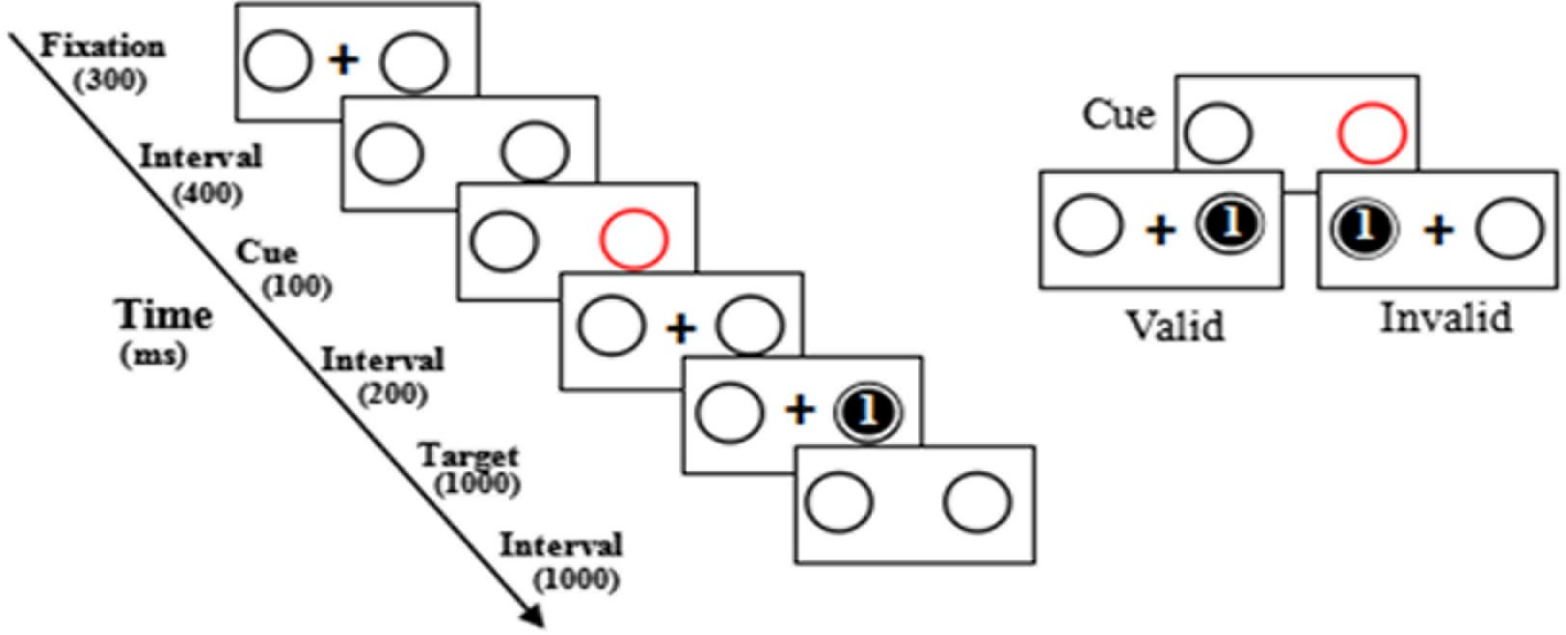
Procedure of the exogenous spatial cuing task of Experiment 2.

The procedure of Experiment 2 was similar to that of Experiment 1, except that the arrow cue was replaced by a single red circle and the interval set as 200 ms(see Fig 3).

#### Design

The design of Experiment 2 was similar to Experiment 1, except that Experiment 2 utilized exogenous attention.

#### Statistical analyses

The statistical analyses of Experiment 2 were the same as Experiment 1.

### Results

The grand mean error rate for all participants was 7.5%, with an average error rate of 10.6% for 7-year-olds, 7.6% for 9-year-olds, 7.8% for 11-year-olds, and 3.9% for adults. Trials with incorrect responses to judgment were excluded from the analysis. Each age group’s mean reaction times (*RT*s) and standard deviations (*SD*) in the endogenous spatial cuing task are shown in Table 7.

**Table 7:** Each age-group’s mean reaction time and standard deviation*(ms)*.

Age-group	The cue	Number 1&2		Number 3&4		Number 6&7		Number 8&9	
		Left hand	Right hand	Left hand	Right hand	Left hand	Right hand	Left hand	Right hand
7-year-old	Valid	726(76)	722(84)	758(67)	741(65)	750(81)	743(83)	786(60)	745(74)
	Invalid	796(88)	771(94)	793(91)	769(99)	813(91)	805(127)	824(81)	805(101)
9-year-old	Valid	642(73)	678(61)	721(70)	674(62)	700(52)	679(70)	725(69)	669(65)
	Invalid	702(69)	730(75)	798(76)	743(91)	751(81)	744(102)	814(93)	754(77)
11-year-old	Valid	619(71)	651(71)	667(69)	638(69)	670(71)	642(75)	705(67)	632(69)
	Invalid	687(75)	738(76)	763(87)	724(85)	736(77)	743(56)	786(69)	724(81)
Adults	Valid	534(66)	568(52)	575(53)	550(56)	570(63)	563(60)	628(63)	548(60)
	Invalid	608(61)	631(64)	680(60)	654(68)	622(65)	655(56)	700(61)	655(59)

An ANOVA approach was used to analyze the SNARC effect(see Table 8.). A significant main effect of age group was found, showing that reaction times decreased as age increased. For example, there was a 76 ms difference between 7-year-olds and 11-year-olds. A significant main effect of hand was found with reaction times of the right hand being shorter than those of the left hand by 19 ms. A significant interaction between cue and age group was found, *F*(9, 330) = 4.82, *p* < .05, η^2^_p_ = .116, and post-hoc comparisons indicated that invalid cues required more time than valid cues in all age groups, and the older age group were faster than all of younger age-groups. The significant interaction between magnitude and hand was found, *F*(3, 330) = 37.36, *p* < .001, η^2^_p_ = .254, and post-hoc comparisons indicated that the RTs for numbers 8 & 9 was shorter than those for 1 & 2.

**Table 8:** Results of ANOVA analyses.

Effect	*df*	*F*	*p*	*η*^*2*^_*p*_
Cue	1	316.68	0.001	0.742
Age-group	3	60.32	0.001	0.622
Cue * Age-group	3	4.82	0.003	0.116
Hand	1	29.05	0.001	0.209
Hand * Age-group	3	0.95	0.417	0.025
Magnitude	3	43.26	0.001	0.282
Magnitude * Age-group	9	1.29	0.243	0.034
Cue * Hand	1	0.69	0.407	0.006
Cue * Hand * Age-group	3	1.79	0.153	0.047
Cue * Magnitude	3	1.22	0.302	0.011
Cue * Magnitude * Age-group	9	2.31	0.016	0.059
Hand * Magnitude	3	37.36	0.001	0.254
Hand * Magnitude * Age-group	9	3.29	0.001	0.082
Cue * Hand * Magnitude	3	2.24	0.084	0.020
Cue * Hand * Magnitude * Age-group	9	0.70	0.711	0.019

The interaction among cue, magnitude, and age group was significant, *F*(9, 330) = 2.31, *p* < .05, η^2^_p_ = .059, and post-hoc comparisons revealed that for valid cues, every age group had a significant magnitude effect. The interaction among hand, magnitude, and age group was significant, *F*(9, 330) = 3.29, *p* < .05, η^2^_p_ = .082, whereby post-hoc comparisons revealed each age group had differences in the hand and magnitude, indicated that the strength of the SNARC effect would be different. The other factors did not exhibit significant main effects or interactions. For example, there was not a significant four-way interaction among cue, hand, magnitude, and age group.

To further investigate the SNARC effect, we used 4(numbers) × 2(hands) ANOVAs for each age group and cue conditions to analyze interactions between magnitude and hand for RTs. Results for each age group are shown in Table 9.

**Table 9:** Results of interaction about magnitude and hand for each age-group in difference cues.

Age-group	Interaction		*df*	*F*	*P*	*η2*_*p*_
7-year-old	Magnitude * Hand	Valid cue	3	1.66	0.182	0.056
		Invalid cue	3	0.05	0.984	0.002
9-year-old	Magnitude * Hand	Valid cue	3	15.10	0.001	0.359
		Invalid cue	3	8.58	0.001	0.241
11-year-old	Magnitude * Hand	Valid cue	3	15.70	0.001	0.386
		Invalid cue	3	7.70	0.001	0.236
Adults	Magnitude * Hand	Valid cue	3	36.98	0.001	0.552
		Invalid cue	3	8.74	0.001	0.226

**Fig 4:**
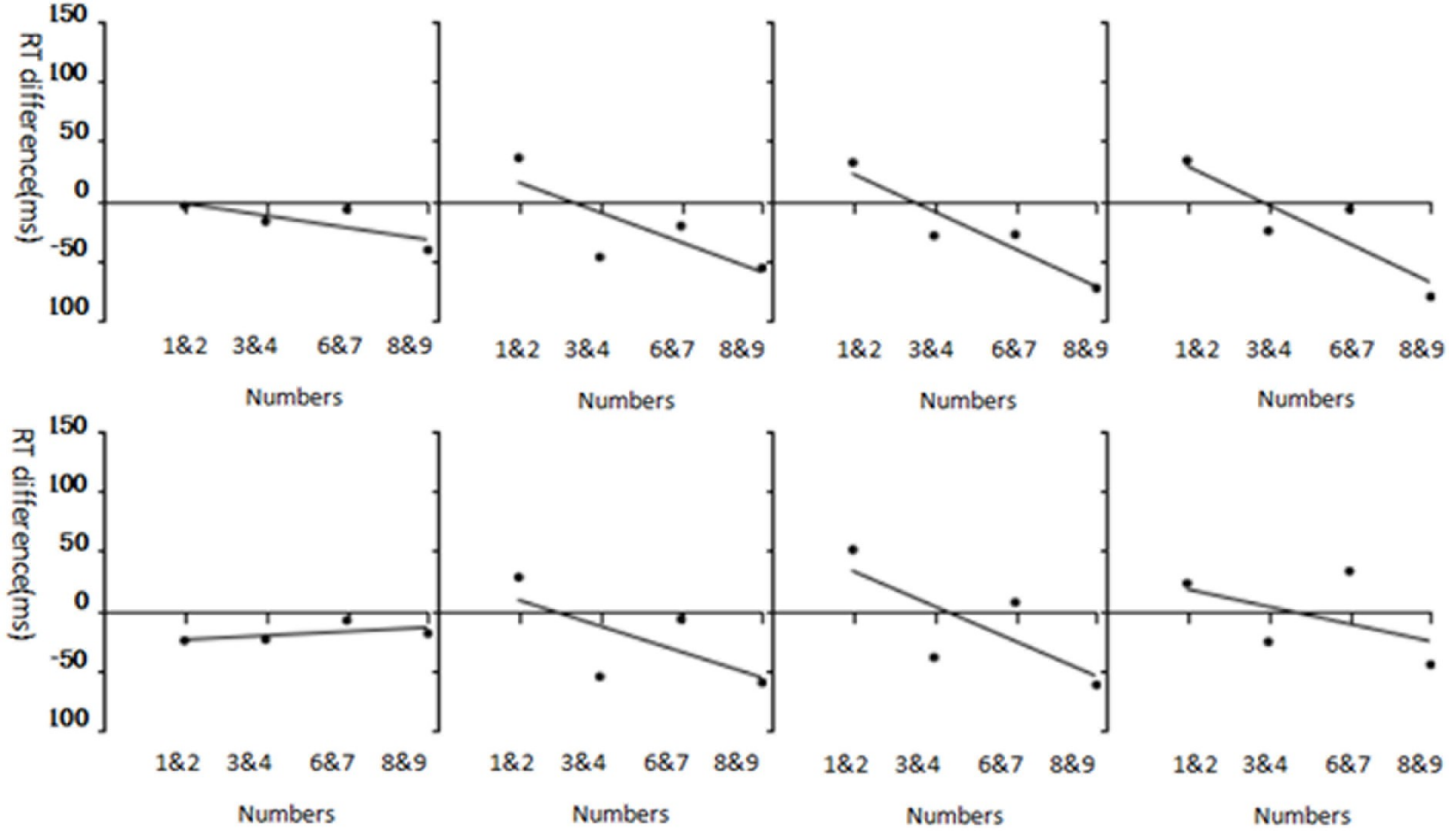
Each age group’s SNARC effect for valid cues (upper panels) and invalid cues (lower panels); from left to right, panels show data for 7-, 9-, 11-year-olds, and adults in each row.

As we can see in Table 8, Hand × Magnitude × Age-group was a significant interaction. When we further investigate the SNARC effect for each age group, we found that the 7-year-olds did not show a significant SNARC effect in either cueing condition (valid cue: *p* >.05, *η2*_*p*_ = 0.056; invalid cue: *p* >.05, *η2*_*p*_ = 0.002) when compared with the standard adults group. As can be seen in Fig 4 and Table 10, all age-groups had significant SNARC effect except for 7-year-olds.

**Table 10:** Results of ANOVA for each age-group in difference cues.

Age-group	Cue	*M*	*SD*	*t*
7-year-old	Valid	-10.28	33.69	-1.64
	Invalid	-11.30	47.84	0.13
9-year-old	Valid	-24.48	27.01	-4.80**
	Invalid	-21.56	31.57	-3.61*
11-year-old	Valid	-31.51	31.99	-5.02**
	Invalid	-29.43	45.31	-3.31*
Adult	Valid	-32.24	23.29	-7.71**
	Invalid	-14.41	31.92	-2.51*

Note.

* *p*<0.05

** *p*<0.01

### Discussion

The results of Experiment 2 revealed that 9-, 11-year-olds, and adults had a significant SNARC effect which was not seen in 7-year-olds in the exogenous attention condition. This is departs from the hypotheses of Experiment 2. These results indicated that the SNARC effect grew stronger from childhood to adulthood. Further, the SNARC effect for all age groups was sensitive to cuing, except for 7-year-olds. This is the most notable difference between the results of Experiment 1 and Experiment 2. Overall, the results of Experiment indicate that exogenous attention could more easily influence the SNARC effect in children.

## General discussion

Both of the present experiments revealed a significant main effect of age, decreasing reaction times with increasing of age in the parity judgment task, and speed of processing that changed by number magnitude. These results are congruent with Wood, Berch, and Dehaene[3,13,20]. The results are also similar to those of Jonidies[36], whereby responses were faster for valid than for invalid trials for each spatial-orienting task. Furthermore, the effect size increased with age, which demonstrates a close correlation between the SNARC effect and age, as the finding as Georges, stronger parity SNARC effects related to better arithmetical math skills in the relatively younger children[63]. At the same time, 9- and 11-year-olds and adults had significant cue effects for both test methods, consistent with Galfano and Rundell[64–65]. These results may be explained by correlations with the development of children’s cognitive processing ability. The current results (Tables 2, 3, 7, and 8) showed that RTs of Chinese children decreased significantly with age. This indicates that they had an understanding of parity, and also reflects increasing efficiency in cognitive processing and response speed with age[66–67]. Furthermore, the main effect of age may be connected to the development of cognition, and from 7- to 11-years-old there was a period of improvement in digital cognitive ability, which tended to be stable at the adult stage[66,68]. Yang et al.[24] and Xu et al.[23] mentioned that Chinese children in kindergarten (mean age 5.8) demonstrated the SNARC effect due to early family training. However, when the spatial orienting of attention added, the results will change. Some families not only teach preschool children counting 1 to 10 (at c. 5.8 years old), but also convey the concept of parity by using “dānshù” and “shuāngshù.” As Zhou et al.[69]noted, Chinese children show an early SNARC effect consistent with automatic magnitude processing due to earlier mathematical acquisition, notably with reference to Chinese pro-education attitudes and early family training[23]. Parity task ability would improve with more profound numerical education and training.

But what role does spatial orienting of attention play? Experiment 1 revealed that each age group exhibited a SNARC effect for valid cues with endogenous attention, and the effect increased from 7- to 11-year-olds for valid cues (Table 4). Note that 9- and 11-year-olds exhibited a SNARC effect for invalid cues according to the repeated measures ANOVA and *t*-test. In contrast, we can see that 11-year-olds behave similarly to adults, as evidenced by the linear fit in Fig 2. Note that prior studies have indicated that endogenous attention is affected more by cognitive load than exogenous attention, even though cognitive resources and meta-cognitive capability improve coping with respect to cognitive load[43–44,36,70]. Moreover, Park and Szũcs suggested that children’s behavioral control abilities are insufficiently mature to counteract interference and facilitation[71–72]. Although children (for example, grade three students) can automatically collect number information, their digital spatial representation abilities are not yet mature, unlike adults who address digital information skillfully and automatically. An invalid cue is inconsistent with the position of the subsequent number, and may interfere with the spatial representation ability due to the child’s use of the mental number line to some extent; thus, they cannot process spatial-representation information effectively. Galfano[64], Rundell and Price [65] reported similar findings. All of these results emphasize the vulnerability of the cue effect, and its dependence on individual psychological-set.

As distinct from endogenous attention, some observation may be made regarding exogenous attention (Experiment 2). First, there was not a significant four-way interaction among age group, cue, hand, and magnitude. Further, in contrast to endogenous attention, 7-year-olds failed to exhibit a SNARC effect (see Tables 9 and 10, and Fig 4). Liu et al. [73] noted that the effects of endogenous and exogenous attention on the SNARC effect were different. The SNARC effect gradually weakens with greater deployment of attention, but cues play a key role in exogenous attention. Because exogenous attention is characterized by bottom-up automated processing, it acts independently of the observer's control. As such, observers cannot easily ignore cues, regardless of whether the information is useful with respect to target location. In contrast, the present study found that valid cue condition was less impactful than the invalid cue condition when comparing the slope of the linear fit for the exogenous attention condition in 9- and 11-year-olds and adults. This indicates that exogenous attention rendered it more difficult to suppress the invalid-cue position, and, thus have profound impact on the SNARC effect. That is, exogenous attention promoted the cue effect and occupied the participants’ attention. This provided a large cognitive load for 7-year-olds.

Another important finding of the current study is that 7-year-olds did not exhibit a SNARC effect for invalid cues in both experiments, which signifies that interference due to invalid cue occurs readily in early childhood. More notably, even for valid cues, there was no SNARC effect for exogenous attention. These results indicate that 7-year-olds are too young to form consistent digital spatial representations and fail to understand the abstract concept clearly. Chinello et al. [74] suggested that such children might not have an adequate conceptual representation, but developmental of finger gnosis (for example, when the use of finger counting) would help improve awareness of associated position in space and emblematic calculation skills, if the children had a high finger gnosis, it means that they would demonstrate a high visuospatial span and high numerical competencies. Thus, when considering the effectiveness of teaching programs, especially in the domain of mathematics, the correlation between finger gnosis and symbolic number processing in early childhood is unclear. Note that extended exercise for counting also can strengthen the relationship between numbers and space, given that studies have revealed that exercise and inhibition ability lead to different SNARC results for different age groups[8,20,75]. However, improved inhibition skills with age would weaken the SNARC effect[75]. The two aspects counterbalance each other, which leads to a stable, continuous SNARC effect. Moreover, Rubinsten, Henik, Berger, and Shahar-Shalev noted that grade one students (c. 6.3 years old) experience difficulties in automatic processing of digital information due to a lack of counting practice, but this situation greatly improves by the end of grade one[57]. In the current experiments, our children were in approximately the middle of grade one, such that limited experience and immature finger gnosis may render the interference effect of invalid cues more significant. Wood et al.’s (2008) meta-analysis of the SNARC effect proposed that greater age could directly impact the SNARC effect via enhancing the habit of associating numbers and space; by 9 years of age, children show an obvious SNARC effect[20].

There is now evidence that SNARC-like effects can be observed in newborn chicks and newborn humans[76–77]. Thus, both biological and cultural factors may be necessary for having spatial-numerical associations. In addition to age-related effects on the SNARC effect, correlations exist between culture and education. As previously mentioned, Berch and colleagues[13] found that the SNARC effect among young children did not emerge until grade 3. However, the onset time of the SNARC effect appeared to be different across cultures, Chinese researchers using the standard parity judgment task to found that Chinese kindergarten had already developed automatic spatial representations of numbers, the results have suggested that perhaps the early development of number cognition and mathematical acquisition was the reason why the spatial representations of numbers show cross-cultural differences[24]. This was attributed to educational and cultural differences in early number learning. For instance, Chinese preschool emphasizes number knowledge to a greater extent than preschools in the United States and the Netherlands[24]. Research that has explored the reasons why children who speaking English are slower in learning the counting sequence compared to Asian children, has theorized that counting practice which including spatially informative cues can facilitate young English-speaking children's learning of the challenging number sequence from 11 to 20[78]. It is key to note that the connection between numbers and space is acquired, and deepened with the improving cognition and learning[79]. Fueyo further highlighted that the relationship between numbers and spatial location may be a reflection of early school education on mental number-line training[80]. With greater relevant training at school, the link between number and spatial location increases. This is supported by Imbo et al.[22], who defined the developmental pattern of verbal and spatial accounts to revealed the SNARC effect, they found that 9- and 11-year-olds in a typical education program showed improved abilities to encode numerical information verbally. Gevers’ study provided a new insight on how language or verbal labeling of spatial information affects numerical encoding tasks [81–82]. In the current study, 9- and 11-year-olds had a clear SNARC effect in invalid-cue conditions, which indicates that their number processing capacity continued to improve in this age period. Note that the current study followed developmental processes in children, and the results revealed that attention and cuing interfere with the cognitive processing of digits in childhood, for both endogenous and exogenous attention.

To summarize, the SNARC effect across all age groups in our study was correlated with endogenous or exogenous attention with respect to cue effects. With increasing age, reaction times decreased and the effect size increased. There were significant main effects of cue in each age group and reaction times for valid cues were shorter than for invalid cues. These findings highlight that 7-year-old Chinese children are clear distracted due to automatic spatial representations of numbers following valid cue, for endogenous attention (i.e., the SNARC effect); however, they did not show a SNARC effect for exogenous attention, regardless of whether the cue was valid or not. As such, the current study highlights that Chinese children’s capability of spatial representations of numbers are influenced by spatial attention and cue effects, 9- and 11-year-olds show a steady SNARC effect under the Posner’s paradigm, especially, the 7-year-olds emerges this capability preliminarily but unsteady.

## Supporting information

S1 File Dataset. (ZIP)

## References

[1] de Hevia M. D., Girelli L., & Macchi C. V. Minds without language represent number through space: origins of the mental number line. Frontiers in Psychology. 2012; 466(3),1–4. 10.3389/fpsyg.2012.00466 PMID:23118732 PMC3484654 23118732

[2] Hoffmann D., Hornung C., Martin R., & Schiltzet C. Developing number–space associations: SNARC effects using a color discrimination task in 5-year-olds. Journal of Experimental Child Psychology. 2013; 116(4), 775–791. 10.1016/j.jecp.2013.07.013 24055929

[3] Dehaene S., Bossini S., & Giraux P. The mental representation of parity and number magnitude. Journal of Experimental Psychology: General. 1993;122(3), 371–396. 10.1037/0096-3445.122.3.371

[4] Dehaene S., Dupoux E., & Mehler J. Is numerical comparison digital? analogical and symbolic effects in two-digit number comparison. Journal of Experimental Psychology Human Perception & Performance. 1990;16(3), 626–641. 10.1037//0096-1523.16.3.626 2144576

[5] Casarotti M., Michielin M., Zorzi M., & Umiltà C. Temporal order judgment reveals how number magnitude affects visuo-spatial attention. Cognition. 2007; 102(1), 101–117. 10.1016/j.cognition.2006.09.001 17046735

[6] Holmes K. J., & Lourenco S. F. When numbers get heavy: is the mental number line exclusively numerical?. Plos One. 2012; 8(3), 1017–1048. 10.1371/journal.pone.0058381 23484023 PMC3590150

[7] Fias W., & Fischer M. H. Spatial representation of number. Journal of Soviet Mathematics. 2005; 43(5), 2662–2663.

[8] Hasher L., & Zacks R. T. Working memory, comprehension, and aging: a review and a new view. Psychology of Learning & Motivation. 1988; 22, 193–225. 10.1016/S0079-7421(08)60041-9

[9] Roettger T. B., & Domahs F. Grammatical number elicits SNARC and MARC effects as a function of task demands. The Quarterly Journal of Experimental Psychology. 2015; 68(6), 1231–1248. 10.1080/17470218.2014.979843 25384199

[10] Shaki S., & Fischer M. H. Reading space into numbers- a cross-linguistic comparison of the SNARC effect. Cognition. 2008; 108(2), 590–599. 10.1016/j.cognition.2008.04.001 18514179

[11] Shaki S., & Fischer M. H. Removing spatial responses reveals spatial concepts-even in a culture with mixed reading habits. Frontiers in Human Neuroscience. 2014; 8(8), 966 10.3389/fnhum.2014.00966 25505403 PMC4244537

[12] Shaki S., Fischer M. H., & Petrusic W. M. Reading habits for both words and numbers contribute to the SNARC effect. Psychonomic Bulletin & Review.2009; 16(2), 328–331. 10.3758/PBR.16.2.328 19293102

[13] Berch D. B., Foley E. J., Hill R. J., & Ryan P. M. D. Extracting parity and magnitude from Arabic numerals: Developmental changes in number processing and mental representation. Journal of Experimental Child Psychology. 1999; 74(4), 286–308. 10.1006/jecp.1999.2518 10552920

[14] Fias W. The importance of magnitude information in numerical processing: evidence from the SNARC effect. Mathematical Cognition.1996; 2(1), 95–110. 10.1080/135467996387552 PMID:28763954

[15] Fischer M. H., & Rottmann J. Do negative numbers have a place on the mental number line. Psychology Science. 2005; 47, 22–32.

[16] Gevers W., Lammertyn J., Notebaert W., Verguts T., & Fias W. Automatic response activation of implicit spatial information: evidence from the SNARC effect. Acta Psychologica. 2006; 122(3), 221–233. 10.1016/j.jecp.2017.04.011 16423320

[17] Zhou X., Chen C., Chen L., & Dong Q. Holistic or compositional representation of two-digit numbers? Evidence from the distance, magnitude, and SNARC effects in a number-matching task. Cognition. 2008; 106(3), 1525–1536. 10.1016/j.cognition.2007.06.003 17707788

[18] Opfer J. E., & Furlong E. E. How numbers bias preschoolers' spatial search. Journal of Cross-Cultural Psychology. 2011; 42(4),682–695. 10.1177/0022022111406098

[19] Schweiter M., Weinhold Zulauf M., & von Aster M. Die Entwicklung räumlicher Zahlenrepräsentationen und Rechenfertigkeiten bei Kindern. Zeitschrift für Neuropsychologie. 2005; 16(2), 105–113. 10.1024/1016-264X.16.2.105

[20] Wood G., Willmes K., Nuerk H. C., & Fischer M. H. On the cognitive link between space and number: A meta-analysis of the SNARC effect. Psychology Science. 2008; 50(4), 489–525.

[21] Van Galen M. S., & Reitsma P. Developing access to number magnitude: A study of the SNARC effect in 7- to 9-year-olds. Journal of Experimental Child Psychology. 2008; 101(2), 99–113. 10.1016/j.jecp.2008.05.001 18602112

[22] Imbo I., Brauwer J. D., Fias W., & Gevers W. The development of the SNARC effect: evidence for early verbal coding. Journal of Experimental Child Psychology. 2012; 111(111), 671–680. 10.1016/j.jecp.2011.09.002 22024386

[23] Xu X., Chen C., Pan M., & Li N. Development of numerical estimation in Chinese preschool children. Journal of Experimental Child Psychology. 2013; 116(2):351–366. 10.1016/j.jecp.2013.06.009 23933179

[24] Yang T., Chen C., Zhou X., Xu J., Dong Q., & Chen C. Development of spatial representation of numbers: a study of the SNARC effect in Chinese children. Journal of Experimental Child Psychology. 2014; 117(1), 1–11. 10.1016/j.jecp.2013.08.011 24121228

[25] Kramer P., Stoianov I., Umiltà C., & Zorzi M. Interactions between perceptual and numerical space. Psychonomic Bulletin & Review. 2011;18(5), 722–728. 10.3758/s13423-011-0104-y 21562926

[26] Lu C. H., & Proctor R. W. The influence of irrelevant location information on performance: a review of the Simon and spatial Stroop effects. Psychonomic Bulletin & Review. 1995; 2(2), 174–207. 10.3758/BF03210959 24203654

[27] Stoianov I., Kramer P., Umiltà C., & Zorzi M. Visuo-spatial priming of the mental number line. Cognition. 2008;106(2), 770–779. 10.1016/j.cognition.2007.04.013 17555739

[28] Zorzi M., & Umiltá C. A computational model of the Simon effect. Psychological Research. 1995; 58(3), 193–205. 10.1007/BF00419634 8570787

[29] Fischer M. H., Castel A. D., Dodd M. D., & Jay P. Perceiving numbers causes spatial shifts of attention. Nature Neuroscience. 2003; 6(6), 555–556. 10.1038/nn1066 12754517

[30] Egeth H. E., & Yantis, S. Visual attention: Control, representation, and time course. Annual review of psychology.1997; 48(48), 269–297. 10.1146/annurev.psych.48.1.269 PMID:14163976 9046562

[31] Theeuwes J. Exogenous and endogenous control of attention: The effect of visual onsets and offsets. Perception & psychophysics. 1991; 49(1), 83–90. 10.3758/BF03211619 2011456

[32] Wolfe J. M., & Horowitz T. S. What attributes guide the deployment of visual attention and how do they do it?. Nature Reviews Neuroscience. 2004; 5(6), 495–501. 10.1038/nrn1411 15152199

[33] Desimone R., & Duncan J. Neural mechanisms of selective visual attention. Neuroscience. 1995; 18(18), 193–222. 10.1146/annurev.ne.18.030195.001205 7605061

[34] Posner M. I. Orienting of attention:Then and now. Quarterly Journal of Experimental Psychology. 1980; 32(1), 3–25. 10.1080/17470218.2014.937446 7367577

[35] Posner M. I., Snyder C. R., & Davidson B.J. Attention and the detection of signals. Journal of experimental psychology. 1980; 109(2), 160–174. 10.1037/0096-3445.109.2.160 7381367

[36] Jonides J. Voluntary versus automatic control over the mind's eye's movement. Attention and performance. 1981; 9(9), 187–203.

[37] Müller H. J., & Rabbitt P. M. Reflexive and voluntary orienting of visual attention: time course of activation and resistance to interruption. Journal of Experimental Psychology: Human Perception and Performance. 1989; 15(2), 315–330. 10.1037//0096-1523.15.2.315 2525601

[38] Nakayama K., & Mackeben M. Sustained and transient components of focal visual attention. Vision research. 1989; 29(11), 1631–1647. 10.1016/0042-6989(89)90144-2 2635486

[39] Hein E., Rolke B., & Ulrich R. Visual attention and temporal discrimination: Differential effects of automatic and voluntary cueing. Visual Cognition. 2006; 13(1), 29–50. 10.1080/13506280500143524

[40] Chica A. B., Bartolomeo P., & Lupiáñez J. Two cognitive and neural systems for endogenous and exogenous spatial attention. Behavioural Brain Research. 2013; 237(1), 107–123. 10.1016/j.bbr.2012.09.027 23000534

[41] Funes M. J., Lupiáñez J., & Milliken B. Separate mechanisms recruited by exogenous and endogenous spatial cues: evidence from a spatial stroop paradigm. Journal of Experimental Psychology Human Perception & Performan. 2007; 33(2), 348–362. 10.1037/0096-1523.33.2.348 17469972

[42] Yeshurun Y., Montagna B., & Carrasco M. On the flexibility of sustained attention and its effects on a texture segmentation task. Vision research. 2008; 48(1), 80–95. 10.1016/j.visres.2007.10.015 18076966 PMC2638123

[43] Berger A., Henik A., & Rafal R. Competition between endogenous and exogenous orienting of visual attention. Journal of Experimental Psychology General. 2005; 134(2), 207–221. 10.1037/0096-3445.134.2.207 15869346

[44] Doallo S., Lorenzo-Lopez L., Vizoso C., Holguin S.R., Amenedo E., Bará S., et al Modulations of the visual N1 component of event-related potentials by central and peripheral cueing. Clinical Neurophysiology. 2005; 116(4), 807–820. 10.1016/j.clinph.2004.11.013 15792890

[45] Corbetta M., & Shulman G. L. Control of goal-directed and stimulus-driven attention in the brain. Nature Reviews Neuroscience. 2002; 3(3), 201–215. 10.1038/nrn755 11994752

[46] Corbetta M., Patel G., & Shulman G. L. The reorienting system of the human brain: from environment to theory of mind. Neuron. 2008; 58(3), 306–324. 10.1016/j.neuron.2008.04.017 18466742 PMC2441869

[47] Peelen M. V., Heslenfeld D. J., & Theeuwes J. Endogenous and exogenous attention shifts are mediated by the same large-scale neural network. Neuroimage. 2004; 22(2), 822–830. 10.1016/j.neuroimage.2004.01.044 15193611

[48] Fischer M. H., & Knops A. Attentional cueing in numerical cognition. Frontiers in Psychology. 2014; 5(5), 1381–1381. 10.3389/fpsyg.2014.01381 25520689 PMC4249257

[49] Goffaux V., Martin R., Dormal G., Goebel R., & Schiltzet C. Attentional shifts induced by uninformative number symbols modulate neural activity in human occipital cortex. Neuropsychologia. 2012; 50(14), 3419–3428. 10.1016/j.neuropsychologia.2012.09.046 23044279

[50] Ranzini M., Dehaene S., Piazza M., & Hubbard E. M. Neural mechanisms of attentional shifts due to irrelevant spatial and numerical cues. Neuropsychologia. 2009; 47(12), 2615–2624. 10.1016/j.neuropsychologia.2009.05.011 19465038

[51] Salillas E., Yagoubi R. E., & Semenza C. Sensory and cognitive processes of shifts of spatial attention induced by numbers: an ERP study. Cortex. 2008; 44(4), 406–413. 10.1016/j.cortex.2007.08.006 18387572

[52] Masson N., & Pesenti M. Attentional bias induced by solving simple and complex addition and subtraction problems. Quarterly Journal of Experimental Psychology. 2014; 67(8), 1–13. 10.1080/17470218.2014.903985 24833320

[53] Dodd M. D. Negative numbers eliminate, but do not reverse, the attentional SNARC effect. Psychological Research.2011; 75(75), 2–9. 10.1007/s00426-010-0283-6 20379741

[54] Jarick M., Dixon M. J., Maxwell E. C., Nicholls M. E. R., & Smilek D. The ups and downs (and lefts and rights) of synaesthetic number forms: validation from spatial cueing and SNARC-type tasks. Cortex. 2009; 45(10), 1190–1199. 10.1016/j.cortex.2009.04.015 19660746

[55] Duncan E. M., & Mcfarland C. E. Isolating the effects of symbolic distance, and semantic congruity in comparative judgments: an additive-factors analysis. Memory & Cognition. 1980; 8(6), 612–622. 10.3758/BF0321378 6163942

[56] Holloway I. D., & Ansari D. Mapping numerical magnitudes onto symbols: the numerical distance effect and individual differences in children's mathematics achievement. Journal of Experimental Child Psychology. 2009; 103(1), 17–29. 10.1016/j.jecp.2008.04.001 18513738

[57] Rubinsten O., Henik A., Berger A., & Shahar-Shalev S. The development of internal representations of magnitude and their association with Arabic numerals. Journal of Experimental Child Psychology. 2002; 81(1), 74–92. 10.1006/jecp.2001.2645 11741375

[58] Pan Y. The experimental study about digital cognitive processing on visual spatial attention. Guizhou People’s Publishing Press 2013.

[59] Rousselle L., & Noël M. P. Basic numerical skills in children with mathematics learning disabilities: a comparison of symbolic vs non-symbolic number magnitude processing. Cognition. 2007; 102(3), 361–395. 10.1016/j.cognition.2006.01.005 16488405

[60] Mclean J. F., & Hitch G. J. Working memory impairments in children with specific arithmetic learning difficulties. Journal of Experimental Child Psychology. 1999; 74(3), 240–260. 10.1006/jecp.1999.2516 10527556

[61] Klein R. M. Inhibition of return. Trends in Cognitive Sciences. 2000;4(4), 138–147. 10.1016/B978-012375731-9/50020-3 10740278

[62] Posner M. I. Components of visual orienting. Attention & Performance. 1984; 32(4), 531–556.

[63] Georges C., Hoffmann D., & Schiltz C. Mathematical abilities in elementary school: do they relate to number-space associations? Journal of Experimental Child Psychology. 2017;161:126–147. 10.1016/j.jecp.2017.04.011 28527362

[64] Galfano G., Rusconi E., & Umiltà C. Number magnitude orients attention, but not against one's will. Psychonomic Bulletin & Review. 2006; 13(5), 869–874. 10.3758/BF03194011 17328387

[65] Rundell R. J., & Price T. D. Adaptive radiation, nonadaptive radiation, ecological speciation and nonecological speciation. Trends in Ecology & Evolution. 2009; 24(7), 394–399. 10.1016/j.tree.2009.02.007 19409647

[66] Kail R. Developmental change in speed of processing during childhood and adolescence. Psychological Bulletin. 1991;109(3),490–501. 10.1037/0033-2909.109.3.490 2062981

[67] Kail R. Processing time, imagery, and spatial memory. Journal of Experimental Child Psychology, 1997; 64(1), 67–78. 10.1006/jecp.1996.2337 9126628

[68] Gibson L. C., & Maurer D. Development of SNARC and distance effects and their relation to mathematical and visuo-spatial abilities. Journal of Experimental Child Psychology. 2016; 150, 301–313. 10.1016/j.jecp.2016.05.009 27376924

[69] Zhou X., Chen Y., Chen C., Jiang T., Zhang H., & Dong Q. Chinese kindergartners' automatic processing of numerical magnitude in Stroop-like tasks. Memory & Cognition. 2007; 35(3), 464–470. 10.3758/BF03193286 17691145

[70] Carrasco M. Visual attention: the past 25 years. Vision Research. 2011; 51(13), 1484–1525. 10.1016/j.visres.2011.04.012 21549742 PMC3390154

[71] Park D. C., Lautenschlager G., Hedden T., Davidson N. S., Smith A. D., & Smith P. K. Models of visuospatial and verbal memory across the adult life span. Psychology & Aging. 2002; 17(2), 299–320. 10.1037/0882-7974.17.2.299 12061414

[72] Szũcs D., Soltész F., Éva Jármi, & Csépe V. The speed of magnitude processing and executive functions in controlled and automatic number comparison in children: an electro-encephalography study. Behavioral & Brain Functions. 2007; 3(1), 1–20. 10.1186/1744-9081-3-23 17470279 PMC1872027

[73] Liu C., & Fu X, L. The influence of attention on the effects of number magnitude in number comparison task. Acta Psychologica Sinica. 2004; 36(3), 307–314.

[74] Chinello A., Cattani V., Bonfiglioli C., Dehaene S., & Piazza M. Objects, numbers, fingers, space: clustering of ventral and dorsal functions in young children and adults. Developmental Science. 2013; 16(3), 377–393. 10.1111/desc.12028 23587037

[75] Wright I., Waterman M., Prescott H., & Murdoch-Eaton D. A new stroop-like measure of inhibitory function development: typical developmental trends. Journal of Child Psychology & Psychiatry & Allied Disciplines. 2003; 44(4), 561–575. 10.1111/1469-7610.00145 12751848

[76] de Hevia M. D., Addabbo M., Nava E., Croci E., Girelli L., & Macchi C. V. Infants' detection of increasing numerical order comes before detection of decreasing number. Cognition. 2017; 158, 177–188. 10.1016/j.cognition.2016.10.022 27835788

[77] Rugani R., Vallortigara G., Priftis K., & Regolin L. Experimental evidence from newborn chicks enriches our knowledge on human spatial–numerical associations. Cognitive Science. 2017; 41(8), 2275–2279. 10.1111/cogs.12523 29023943

[78] Dunbar K., Ridha A., Cankaya O., Lira C. J., & Lefevre J. A. Learning to count: structured practice with spatial cues supports the development of counting sequence knowledge in 3-year-old English-speaking children. Early Education & Development. 2017; 28, 308–322. 10.1080/10409289.2016.1210458

[79] Ninaus M., Moeller K., Kaufmann L., Fischer M. H., Nuerk H. C., & Wood G. Cognitive mechanisms underlying directional and non-directional spatial-numerical associations across the lifespan. Frontiers in Psychology. 2017; 8, 14–21. 10.3389/fpsyg.2017.01421 PMID:28878716 28878716 PMC5572383

[80] V Fueyo D. B. Using number line procedures and peer tutoring to improve the mathematics computation of low-performing first graders. Journal of Applied Behavior Analysis. 1998; 31(3), 417–430. 10.1901/jaba.1998.31-417 PMCID: PMC1284138

[81] Gevers W., Santens S., Dhooge E., Chen Q., Van d. B. L., & Fias W., et al Verbal-spatial and visuo-spatial coding of number-space interactions. Journal of Experimental Psychology General. 2010; 139(1), 180–190. 10.1037/a0017688 20121318

[82] Simon O., Kherif F., Flandin G., Poline J. B., Riviere D., Mangin J. F., et al Automatized clustering and functional geometry of human parietofrontal networks for language, space, and number. Neuroimage. 2004; 23(3), 1192–1202. 10.1016/j.neuroimage.2004.09.023 15528119

